# Possible role of the gut microbiota in the pathogenesis of anorexia nervosa

**DOI:** 10.1186/s13030-021-00228-9

**Published:** 2021-11-29

**Authors:** Nobuyuki Sudo

**Affiliations:** Department of Psychosomatic Medicine, Graduate School of Medical Sciences, Kyushu University, 3-1-1 Maidashi, Higashi-ku, Fukuoka, 812-8582 Japan

**Keywords:** Anorexia nervosa, Eating disorder, Gut microbiota, Hyperactivity, Uremic toxin

## Abstract

Anorexia nervosa (AN), an eating disorder, is characterized by extreme weight loss and fear of weight gain. Psychosocial factors are thought to play important roles in the development and progression of AN; however, biological factors also presumably contribute to eating disorders. Recent evidence has shown that the gut microbiota plays an important role in pathogenesis of neuropsychiatric disorders including AN. In this article, we describe the possible role of the gut microbiota in the development and persistence of AN, based on the latest research works, including those of our group.

## Introduction

More than 1000 species of microbes are present in the human gut. They are collectively called the gut microbiota and are involved in various host functions [1–3]. Accumulated evidence suggests that gut microbes can play a role not only in regulating body weight [4] but also in the development and exacerbation of neuropsychiatric diseases [5–9].

Anorexia nervosa (AN) is an eating disorder characterized by extreme weight loss and a fear of weight gain [10, 11]. In general, psychosocial issues are reported to play important roles in the pathology of AN [12–14]; however, biological factors are also presumed to contribute to this pathological process [15, 16]. Recently, the gut microbiota has emerged as an important factor affecting AN pathogenesis.

In the first two sections of this article, we review a historical view that recognizes the commensal microbiota as an important factor affecting weight control and behavioral characteristics. Then, we discuss recent topics about this theme based on the latest research works, including those of our group. Unlike comprehensive systematic reviews and metanalyses [17, 18] that have been published recently, our aim is to use information from existing literature to generate testable hypotheses regarding the role of the gut microbiota in AN pathogenesis. By doing so, we hope to encourage researchers to conduct cause-effect studies about eating disorders based on realistic mechanistic hypotheses.

### Gut microbes exert a significant impact on body weight gain

The fact that gut bacteria are involved in weight control and growth is not a novel finding, but is a long known basic theory in livestock industries. For example, antibiotics have often been used to promote livestock growth [19, 20], called antibiotic-induced growth promotion (AIGP). The use of antibiotics for AIGP is still being exploited in several countries, including the US and Japan. The precise mechanism for AIGP is unclear, but the fact that growth promotion is absent when administering antibiotics to germ-free animals deficient in bacteria [21, 22], indicates that the gut microbiota plays a critical role in this promoting effect [20]. Gordon et al. [23–25] used an elegant method of fecal transplantation to demonstrate that the gut microbiota is critically important for regulating body weight. Furthermore, another group [26] showed that transplantation of *Christensenella minuta* into mice with an obese phenotype suppressed weight gain. Thus, these findings indicate that the gut microbiota can affect body weight regulation by modulating the gut microbial ecology.

### Commensal microbes affect not only host stress response but also host behaviors

When exposed to stress stimuli, the hypothalamic-pituitary-adrenal (HPA) axis is activated to maintain homeostasis of the body [27]. Interestingly, the HPA axis is known to be affected by both genetic determinants and postnatal environmental factors during infancy [28, 29]. Because gut microbes are an important environmental factor, we hypothesized that the gut microbiota plays a role in host stress responses. In 2004, we first demonstrated that gut microbes determine the HPA response to stressors, using germ-free (GF) and gnotobiotic mice [30]. The degree of plasma ACTH and corticosterone elevation in response to 1-h restraint stress was higher in GF mice than in specific pathogen free (SPF) mice. Furthermore, as summarized in Table 1, mono-association with *B. infantis*, a representative inhabitant of the neonate gut, decreased the HPA stress response to SPF, while mono-association with *Bacteroides vulgatus* had no effect. The hormonal stress response of rabbit-derived enteropathogenic *Escherichia coli* (EPEC)-monoassociated mice was substantially higher than that of GF mice, although such an exaggerated response was not found in mice with an EPEC mutant strain, ΔTir [31], which was uninternalized due to defects in the translocated intimin receptor.

**Table 1:** Effects of restraint stress (RS) on plasma ACTH and the corticosterone levels of gnotobiotic mice

	ACTH (pg/ml)		Corticosterone (ng/ml)	
	Basal	1 h-RS	Basal	1 h-RS
GF	66 ± 12	188 ± 16	19 ± 3.9	131 ± 12
SPF	54 ± 6.1	106 ± 20***	21 ± 6.5	86 ± 9.9***
*B. infantis*	60 ± 9.8	113 ± 15***	21 ± 5.2	79 ± 9.5***
*B. vulgatus*	63 ± 9.9	166 ± 14	17 ± 6.8	140 ± 14
EPEC	49 ± 15	243 ± 22*	19 ± 6.6	172 ± 20*
ΔTir	60 ± 9.5	153 ± 25	15 ± 3.6	102 ± 17

Plasma ACTH and corticosterone levels were measured before or immediately after 1 h of RS in germ-free (GF), specific-pathogen-free (SPF), and gnotobiotic mice reconstituted with a single strain with *Bifidobacterium infantis* (*B. infantis*), *Bacteroides vulgatus* (*B. vulgatus*), rabbit-derived enteropathogenic E-coli (EPEC), or EPEC mutant strain (ΔTir) at 9 wks of age, as described previously [30]. *** *P* < 0.001 and * *P* < 0.05 (significantly different from the GF value)

The finding that commensal bacteria can substantially affect the HPA stress response later in life raises an interesting question about whether gut bacteria can change host behavior. This question has been addressed in the past decade by several independent groups, including our own [32–37]. The commensal microbiota is a crucial factor in modulating the host’s behavioral profile. For example, we developed a reliable method to accurately analyze the behavior of GF mice maintained in an isolator [35]. This method enabled us to evaluate GF animal behavior without the risk of exposure to contamination. Using this system, GF mice were found to be more active and anxious than EX-GF mice that were transplanted with a normal SPF microbiota, based on open-field and marble-burying tests (Table 2). Colonization with *B. infantis* decreased the locomotor activity to the EX-GF level but had little effect on the anxiety levels. In contrast, mono-association with *Clostridium coccoides* reduced anxiety levels; however, it did not affect locomotor activity [35].

**Table 2:** Transplantation of normal gut microbiota renders germ-free (GF) mice less active and anxious

		7 wks of age	10 wks of age	16 wks of age
GF	OFT (DT30)	62.7 ± 12.2	63.7 ± 9.4	66.4 ± 21.4
	MBT (NBM)	14.3 ± 5.3	15.9 ± 5.9	14.6 ± 6.2
EX-GF	OFT (DT30)	46.5 ± 7.1***	54.0 ± 8.9**	53.5 ± 11.3*
	MBT (NBM)	12.5 ± 5.8	12.4 ± 4.5*	9.5 ± 5.5*

Parent GF mice were transplanted with feces of SPF mice, and their offspring were used as EX-GF mice, as previously described [35]. GF and EX-GF mice at 7, 10 and 16 wks of age were subjected to the open-field test (OPT) and marble-burying test (MBT). The total distance traveled for 30 min (DT30; meter) was automatically calculated as spontaneous locomotor activity. The number of marbles buried for 30 min (NBM) was counted as a parameter of anxiety-like behavior. All data are expressed as mean ± SD. *p < 0.05, **p < 0.01 and ***p < 0.001 (significantly different from the corresponding GF value)

Collectively, these results indicate that gut microbiota can exert a substantial effect on the behavioral phenotype as well as the ability of the host to respond to stressors.

### Possible relation between gut microbiota and AN pathologies

Patients with AN who have extremely low body weight often show marked resistance to weight gain in response to calorie intake; therefore, they usually require more calories to increase body weight than healthy subjects with normal weight [38–40]. Several factors such as increased physical activity [41] and diet-induced thermogenesis [42] are suggested to be involved in this phenomenon; however, the precise mechanisms for this are largely unknown. In addition, other important complications of AN include comorbid anxiety and depressive disorders. A well-known study, *“the Minnesota Starvation Experiment”* [43], which was performed during World War II, clearly showed that starvation significantly affected both physical and psychological conditions [43, 44]. Thirty-six volunteers who refused military service for religious reasons participated in this study. They were psychologically resilient before the experiment; however, most experienced periods of severe emotional distress during the study. This indicates that some psychiatric symptoms found in patients with AN could be explained by the effects of starvation. However, how and to what extent starvation affects the psychological and social functioning of patients with AN remain largely unknown.

Considering the emerging effects of gut microbes on body weight control or behavioral phenotypes, we speculated that commensal bacteria may play an important role in the onset and exacerbation of AN. Undoubtedly, patients’ premorbid traits and psychosocial stressors are critical to the onset of AN. Prolonged physiological and psychological stress, including severe weight loss and extreme dietary habits, may influence the composition of the gut microbiota and subsequently induce “gut dysbiosis.” This disturbed bacterial community may contribute to a decrease in food efficiency or behavioral abnormalities. For example, hyperactivity, one such abnormal behavior, may further strengthen the resistance to weight gain.

### Gut dysbiosis in patients with AN

Several researchers have investigated whether gut dysbiosis exists in the gut of patients with AN and have shown that patients with AN show “dysbiosis”, abnormal features of gut microbiota [45–48]. Armougom et al. [45] reported that *Methanobrevibacter smithii* was more frequently detected in patients with AN than those with low body weight due to diseases other than AN. Interestingly, these methanogenic archaea are often found in patients with constipation-predominant irritable bowel syndrome [49, 50]. Mack et al. [47] found that the ratio of mucin-degrading bacteria to *Clostridium* cluster I, XI, and XVIII in an AN group was higher and the butyric acid-producing genus *Roseburia* was lower than that of the intestinal flora of normal body weight subjects. In addition, the proportion of *Bacteroidetes* phylum in patients with AN was significantly reduced compared to normal-weight subjects and did not recover after weight gain. Our group [48] used the intestinal bacterial analysis system YIF-SCAN® that was developed by Yakult Central Laboratory to examine the intestinal bacteria of female subjects with AN and age-matched healthy women. The total bacterial, *Clostridium coccoides*, *Clostridium leptum*, *Bacteroides fragilis,* and *Streptococcus* counts were significantly lower in the AN group than the control group. The detection rate of the *Lactobacillus plantarum* subgroup was significantly lower in the AN group than in the control group. Based on 16S rRNA sequencing methods, we also reported a lower relative abundance of the phylum *Bacteroidetes* in patients with AN than healthy age-matched controls [51]. As summarized in Table 3, several recently published papers [52–57] have also shown changes in gut microbial ecology at the phylum or genus levels; however, no specific bacteria were identified among these reports. Thus, although the results differ depending on the patient’s background or analytical methods, “gut dysbiosis” is consistently observed in patients with AN.

**Table 3:** Gut microbial composition of patients with anorexia nervosa (AN)

Characteristics of AN participants	Methods	Main findings	^**1**^Country	^**2**^Year	^**3**^Ref
AN (*n* = 9, age 19–36 years, ^4^BMI 12.73 ± 1.6)	^5^PCR	*Methanobrevibacter smithii* concentration was higher in AN patients than in lean control.	France	2009	[45]
AN (*n* = 14, age 27.3 ± 10.8, BMI median 13.5)	PCR	*Firmicutes, Bacteridetes, Methanobrevibacter smithii,* and *E. coli* were found in 98.5, 67, 64 and 51% of the participants, respectively.	France	2013	[52]
AN (*n* = 16, Age 28 ± 11.7 years, BMI 16.2 ± 1.5)	^6^16S rseq	Alpha diversity was lower in AN patients than in healthy controls. At genus level, *Anaerostipes* and *Faecalibacterium* were reduced versus a healthy comparison group.	USA	2015	[46]
^7^ANR (n = 14, Age 28.1 ± 10.7 years, BMI 12.7 ± 1.5), ^8^ANBP (*n* = 11, Age 32.5 ± 9.4 years, BMI 13 ± 1.2)	PCR	Amounts of *Clostridium coccoides* group, *Clostridium leptum* subgroup, *Bacteroides fragilis* and *Streptococcus* were lower in AN patients than in healthy controls.	Japan	2015	[48]
AN (*n* = 55, Age 23.8 ± 6.8 years, BMI 15.3 ± 1.4)	16S rseq	The ratio of mucin-degrading bacteria to *Clostridium* cluster I, XI, and XVIII was increased, while the butyric acid-producing genus *Roseburia* was decreased, relative to controls.	Germany	2016	[47]
AN (*n* = 15, BMI 13.9 ± 2.1)	16S rseq	*Enterobacteriaceae* and *Methanobrevibacter smithii* levels were increased compared with healthy controls; while, the genera *Roseburia*, *Ruminococcus* and *Clostridium*, were depleted.	Italy	2017	[53]
AN (*n* = 18, Age 22.4 ± 3.2 years, BMI 15.3 ± 1.3)	16S rseq	Only *Coriobacteriaceaeone* levels were significantly enriched in AN compared to other groups.	Austria	2017	[54]
AN (*n* = 17, Age 21.8 ± 3.6 years, BMI 15.2 ± 1.3)	16S rseq	*Ruminococcaceae* and *Faecalibacterium* were increased in a low-zonulin population. No specific comments about AN.	Austria	2018	[55]
AN (*n* = 33, Age: 32 ± 12, BMI 11.7 ± 1.5)	16S rseq	*Klebsiella* and *Salmonella* levels were more abundant in AN patients whereas *Eubacterium* and *Roseburia* were significantly less abundant in patients than controls.	France	2019	[56]
ANR (*n* = 10, Age 25 ± 2.8 years, BMI 13.3 ± 0.3)	16S rseq	A lower relative abundance of phylum Bacteroidetes was observed in AN in comparison to age-matched healthy women.	Japan	2019	[51]

^1^Country, where each study was conducted; ^2^year, when each study was reported; ^3^ref, references number; ^4^BMI, body-mass index; ^5^PCR, polymerase chain reaction using targeted-bacteria specific primers; ^6^16S rseq, 16S rRNA sequencing analysis; ^7^ANR, AN restricting-type; ^8^ANBP, AN binge-purging type

### Can gut dysbiosis contribute to AN pathologies: analyses using gnotobiotic animal models

The existence of gut dysbiosis does not always relate to impaired functional outcomes; namely, such disturbed microbiota may not be a causal factor contributing to AN pathologies but an epiphenomenon resulting from lengthy starvation. To test this, we transplanted the gut microbiota of patients with AN into GF mice to establish gnotobiotic mice (gAN) whose microbiota consisted of the intestinal microbiota of patients with AN [51]. The gAN mice showed a significantly decreased weight gain compared with the gnotobiotic mice (gHC) transplanted with the gut microbiota of healthy women. Similarly, food efficiency (weight gain/food intake) was also lower in the gAN mice than in the gHC mice. Moreover, the gAN mice also exhibited increased anxiety-like behaviors relative to the gHC mice, when evaluated by open field and marble-burying tests. Interestingly, marble-burying behaviors displayed the highest correlation with the relative abundance of the genus *Bacteroides*. Moreover, the administration of *Bacteroides vulgatus*, which belongs to the genus *Bacteroides* and is a predominant species of the *Bacteroides fragilis* group in adult humans [58], reversed the behavioral abnormalities in the gAN mice.

These results indicate that some characteristic features of patients with AN can be reproduced by transplanting the AN gut microbiota. Bacteria, such as *Bacteroides vulgatus,* may play a protective role against the development of pathologies specific to patients with AN. Recently, Glenny et al. [59] reported that fecal transplantation from patients with AN exerted no significant effects on body weight in GF mice. A precise reason for this discrepant result is unclear; however, this may be related to the methodology of fecal transplantation, which involved the use of frozen feces, as they suggested. Therefore, the results of our study, which utilized fresh feces, should be validated by future experiments using a larger number of mouse colonies and different human donors.

### Molecules potentially involved in AN pathology: analyses using serum metabolome profiles

The above results based on animal experiments provide valuable information about some pathological features of AN; nonetheless, they are inapplicable to human conditions. Therefore, we performed metabolome analyses using human materials.

#### Uremic toxins and related compounds

We detected 275 metabolites in serum samples from patients with AN and healthy controls [60]. Although the patients with AN enrolled in this study failed to show any apparent renal dysfunction, the serum levels of guanidinosuccinic acid and *N2*-phenylacetylglutamine, a uremic toxin, were significantly higher in the patients with AN restricting type (ANR). Therefore, precise quantification of uremic toxins was performed using selected-reaction monitoring of liquid chromatography/electrospray ionization-mass spectrometry/mass spectrometry at Kureha Corporation, as reported [61–63]. The serum p-cresol (PCS), indole-3-acetic acid, and phenyl sulfate levels were significantly higher in an AN group than in an age-matched control group [60]. Because these uremic toxins are produced by gut microbes, gut microbes may contribute to increased uremic toxins. This possibility was supported by our results showing that serum PCS levels in an ANR group (but not a control group) correlated positively with the abundance of a *Clostridium coccoides* group or a *Clostridium leptum* subgroup [60]. Interestingly, in another cohort of ANR patients [51], the relative abundance of members of the genus *Blautia* was significantly higher in ANR patients than in age-matched healthy women. Previously, it was reported that the genus *Blautia* was the most abundant subgroup in human intestinal *Clostridium coccoides* group populations that were identified using the YIF-SCAN system [64]. These findings suggest that increased populations of certain bacteria belonging to the genus *Blautia* may help elevate the serum uremic toxin levels of patients with ANR.

Yokoyama et al. [65] reported that PCS derived from gut microbes may slow the growth of weanling pigs and that inhibiting PCS production with antibiotics may be one of the mechanisms by which antibiotics can induce AIGP [20]. The compound was also found to play a role in the development and progression of neuropsychiatric diseases, such as autism [66–68]. For example, Hsaio et al. [69] suggested that 4-ethylphenylsulfate, a sulfated compound of PCS, may promote the development of autistic-like behavior in a mouse model with maternal immune activation. Nonetheless, whether PCS or its related compounds can affect weight gain and behavioral characteristics of patients with AN remains unclear.

#### Amino acids: tryptophan and related molecules may be key players responsible for AN-specific pathologies

Serum metabolomic measurement showed another interesting finding regarding AN. Serum levels of 10 amino acids, including asparagine, tyrosine, isoleucine, alanine, histidine, leucine, methionine, proline, tryptophan (TRP), and valine, were lower in patients with AN than age-matched healthy women, when the false-discovery rate corrected *p*-value was set at less than 0.1. According to pathway enrichment analyses, the pathway of “phenylalanine, tyrosine, and TRP biosynthesis” was ranked as the highest impact score [60].

Since Kaye’s pioneering works [70, 71], TRP has been demonstrated to play an important role in the development and maintenance of AN. In fact, in malnourished and emaciated individuals with AN, reduced plasma TRP availability [72–74] and reduced 5-hydroxyindoleacetic acid levels in the CSF have been reported [71]. This decreased serotonergic system may be involved in AN pathology by changing the host behavior. Indeed, increased locomotor activity, hyperactivity, is often seen in subjects with AN and has been regarded as a key characteristic of the disorder [75–77]. Interestingly, Uchida et al. [78] reported that mice fed TRP-limited diets exhibited increased locomotor activity. This indicates that a dearth of tryptophan due to decreased dietary intake can exacerbate AN-specific behavioral abnormalities, such as hyperactivity, via modulating the brain 5-HT system, subsequently aggravating poor weight gain by increasing exercise-induced calorie consumption. This series of events eventually forms a vicious cycle, which may substantially contribute to the perpetuation of AN pathology.

### Future perspectives

Increasing attention has been paid to the role of gut microbiota in AN. However, in terms of gut microbes, the following questions remain to be addressed.

1. There is a pressing need to develop more effective therapeutics for adult patients with AN who are refractory to usual treatments. Probiotics may be a useful adjunctive therapy to achieve better weight gain and maintain appropriate mental conditions. This is clinically important and should be addressed by randomized controlled studies with large sample sizes.
2. Some gut bacteria metabolize indigestible dietary fiber or oligosaccharides and produce short chain fatty acids (SCFAs), such as acetate, propionate, and butylate [79]. SCFAs are now considered to be one of the key molecules that can affect neuropsychiatric functions [5, 80]. Such bacteria-generated SCFAs exert an anxiolytic effect in mice [81, 82]; therefore, pre- or pro-biotic interventions that have an ability to increase fecal SCFAs may be a therapeutic option to improve mental conditions in patients with AN.
3. The majority of gut bacteria resides in the colon; therefore, the colonic bacteria have been extensively studied. However, how and to what extent microbes residing in the jejunum or ileum can contribute to host pathophysiology are largely unknown and should be clarified. For example, small intestinal bacterial overgrowth has been found to occur under some pathological conditions [83], and because amino acids, including tryptophan, are usually absorbed via transporters present in the upper GI tract [84, 85], it is theoretically possible that bacteria in the small intestine may play a role in the development and exacerbation of AN pathology by modulating the ability of the microbes to metabolize diet- or host-derived proteins. These bacteria may confer protection against AN-specific behavioral abnormalities by producing amino acids such as tryptophan.
4. Recently, some species of gut microbes have been reported to synthesize d-amino acids as well as l-amino acids [86, 87]. D-serine is known to activate the N-methyl-D-aspartic acid receptor as its co-agonist [88]. Therefore, D-amino acids, such as d-tryptophan in the gut lumen, may exert a substantial effect on brain function similar to D-serine.

**Fig. 1:**
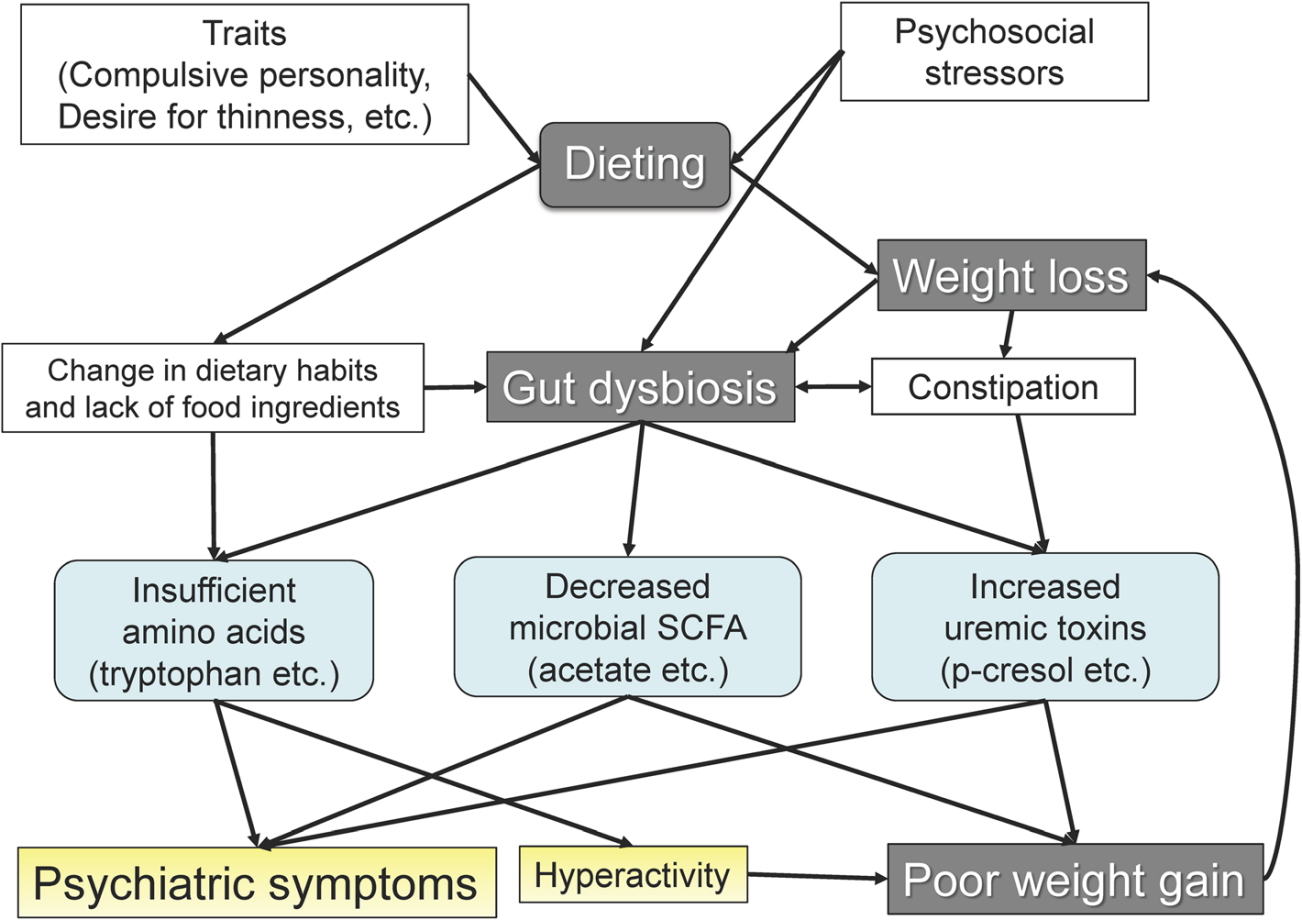
Possible role of the gut microbiota in the pathogenesis of anorexia nervosa (AN). Premorbid traits, such as compulsive personality, perfectionism, and drive for thinness, and psychosocial stressors play a crucial role in AN development [74]. Consequently, changes in dietary habits and altered intake of food ingredients, induced by dieting, lead to a dearth of essential amino acids [60] and “gut dysbiosis” [45–48, 51–56]. Physical and psychological stresses accompanying extreme weight loss may also affect the disturbed microbial ecology of the gut [89, 90]. Moreover, gut dysbiosis, together with the stresses, also induces constipation by impairing gut motility function [49, 50]. Prolonged constipation with gut dysbiosis elevates uremic toxins [60], such as p-cresol, which is possibly involved in poor weight gain [65] and psychiatric symptoms [69]. Decreased short chain fatty acids (SCFA), especially acetate, derived from gut dysbiosis [48] may also contribute to impaired weight control [79] and persistent anxiety [81, 82]. Finally, hyperactivity, a key characteristic of AN [75–77], is potentially induced by an insufficient intake of essential amino acids, such as tryptophan, and further perpetuates low body weight via increasing calorie expenditure

Figure 1 shows our working hypothesis concerning the possible role of the gut microbiota in the development and perpetuation of AN.

## Conclusion

The theory of “autointoxication,” which states that toxins generated in the gut exert a negative impact on brain function, inducing depression, anxiety, and other mental diseases [91–93], was long regarded as an irrational concept. Only recently has scientific research been conducted on the topic, and it has become extensively studied. Further developments in this field could elucidate the role of gut microbes in the pathogenesis of eating disorders and further provide a strong rationale for probiotic intervention as a treatment for patients with AN.

## Data Availability

Not applicable.
